# Effect of Repeated Simulated Disinfections by Microwave Energy on the Complete Denture Base Adaptation

**DOI:** 10.2174/1874210600802010061

**Published:** 2008-04-29

**Authors:** Rafael L.X. Consani, Rose Y Iwasaki, Marcelo F Mesquita, Wilson B Mendes, Simonides Consani

**Affiliations:** 1 Department of Prosthodontics and Periodontics, Piracicaba Dentistry School, State University of Campinas, Piraci-caba, SP, Brazil; 2 Under Graduate Student, Piracicaba Dentistry School, State University of Campinas, Piracicaba, SP, Brazil; 3 Department of Prosthodontics and Periodontics, Piracicaba Dentistry School, State University of Campinas, Piracicaba, SP, Brazil; 4 Department of Clinics, Dentistry School of the Itauna University, Itauna, MG, Brazil and; 5 Department of Restorative Dentistry, Piracicaba Dentistry School, State University of Campinas, Piracicaba, SP, Brazil

**Keywords:** Denture base, adaptation, microwave disinfection, flask closure

## Abstract

This study evaluated the effect of repeated microwave disinfections on the adaptation of the maxillar denture base using 2 different flask closure methods. Twenty stone cast-wax base sets were prepared for flasking by traditional cramp or RS system methods. Five bases for each method were submitted to 5 repeated simulated disinfections in a microwave oven with 650W for 3 minutes. Control bases were not disinfected. Three transverse cuts were made through each stone cast-resin base set, corresponding to canine, first molar, and posterior region. Measurements were made using an optical micrometer at 5 points for each cut to determine base adaptation: left and right marginal limits of the flanges, left and right ridge crests, and midline. Results for base adaptation performed by the flask closure methods were: traditional cramp (non-disinfected = 0.21 ± 0.05mm and disinfected = 0.22 ± 0.05mm), and RS system (non-disinfected = 0.16 ± 0.05 and disinfected = 0.17 ± 0.04mm). Collected data were submitted to ANOVA and Tukey test (α=.05). Repeated simulated disinfections by microwave energy did not cause deleterious effect on the base adaptation, when the traditional cramp and RS system flask closure methods were compared.

## INTRODUCTION

In addition to the changes occurred due to several variables, such as linear shrinkage of the acrylic resins [1], denture processing [2,3], commercial types of acrylic resins [4], flask closure methods [5], and post-pressing times [6], treatments for denture disinfection performed by chemical [7] and microwave irradiation methods can also promote linear dimensional changes [8-1].

Prosthesis can be contaminated by microorganisms during manufacture or manipulation, or by the patients. As an effort to eliminate or decrease cross-contamination, chemical solutions should be used for prosthesis disinfection. Materials sent from dental clinics to prosthetic laboratories were contaminated by bacteria [12], and sterile prostheses could be contaminated during polishing or by microorganisms transferred from other prostheses during laboratory practice [13-15].

Glutaraldehyde, sodium hypochlorite, iodoform, or chlorine dioxide had been suggested for prosthesis chemical disinfection to avoid cross-contamination, [16-21]; however, this method shows disadvantages such as prosthesis staining and oral tissue reactions [22, 23].

Microwave energy was used for polymerization of the thermally activated acrylic resins [24], and microwave irradiation of resilient materials and acrylic resins in a microwave oven effectively sterilized specimens contaminated by fungi [22], *Candida albicans *or* Staphylococcus aureus* [23]. Microwave energy has been recommended for prosthesis disinfection [25] when the probability of the denture base being contaminated internally and externally is considered [21].

Earlier study showed comparison between chemical disinfection with glutaraldehyde solution and by microwave energy (500W intensity for 3 or 15 minutes) on hardness, dimensional changes, and flexural strength of the acrylic resin. The findings showed that these properties were not altered by either disinfection procedures [25].

Some studies have identified whether microwave disinfection promotes changes in the complete denture base adaptation [8-1, 26]. Although a soft tissue displacement of only 0.25mm would be necessary to allow almost complete seating of the denture on the oral tissue [27], disinfection procedures should not cause dimensional changes or distortion in the denture base, since these factors can compromise the retention and stability of the dentures.

The purpose of this study was to evaluate and compare the effect of repeated simulated microwave disinfections on the adaptation of the maxillary complete denture achieved when bases were prepared using the traditional cramp flask closure (TFC) and Restriction System flask closure (RSFC) methods [28]. The research hypothesis tested in this *in vitro* study was that the denture base adaptation prepared using the TFC and RSFC methods could be adversely affected by repeated simulated disinfections by microwaves energy.

## MATERIAL AND METHODS

### Material

An acrylic resin (Batch # 009-04, Classico; Classico Dental Products, Sao Paulo, SP, Brazil) was used to fabricate maxillary complete denture bases. The manufacturer purports that Classico is a conventional acrylic resin based on a polymethyl methacrylate copolymer with heat activation (Powder: prepolymerized spheres of polymethyl methacrylate and benzoyl peroxide as initiator. Liquid: unpolymerized copolymer of methyl methacrylate and ethylacrylate, and hydroquinone as inhibitor).

### Methods

Impressions were made from a metal die simulating an edentulous maxillary arch with vinylpolysiloxane duplicating material (Batch # 37608, Elite Double 8; Zhermack, Rovigo, Italy), and twenty correspondent stone casts were poured in type III dental stone (batch # 00709, Herodent; Vigodent, Rio de Janeiro, RJ, Brazil). A 2-mm thick wax base (Batch # 195-05, Epoxiglass; Epoxiglass Chemical Products, Diadema, SP, Brazil) was made by the same technician for each cast stone and measured with a caliper for standardization purposes (Golgran; Colgran Dental Products, Sao Paulo, SP, Brazil). The stone cast-wax base sets were codified with numbers to blind the examiner, and randomly divided into the following groups (n=5): (1) bases made for the traditional cramp flask closure method (TFC) and non-disinfected (ND); (2) bases made for the TFC method and submitted to repeated simulated disinfections by microwave (RSD); (3) bases made for the RS system flask closure method (RSFC) and ND; and (3) bases made for the RSFC method submitted to RSD.

The stone cast-wax pattern sets were flasked in the lower part of traditional brass flasks (Safrany; J. Safrany Mettalurgy, Sao Paulo, SP, Brazil) with type II dental plaster (Batch # 2410, Star; Chaves, Fortaleza, CE, Brazil, and type III dental stone (Herodent; Vigodent) was used in the upper portion. After 1 hour, the flasks were placed in boiling water to soften and remove the wax pattern. After removal, the stone was cleaned with boiling water and liquid detergent solution (Limpol; Bombril-Cirio, Sao Paulo, SP, Brazil). Sodium alginate (Batch # 998010, Isolak; Classico Dental Products) was used as a mold separator.

The acrylic resin (Classico; Classico Dental Products) was prepared using a solution with a ratio of 35.5g powder to 15mL liquid, according to the manufacturer’s instructions. In the TFC groups (ND and RSD), the flasks were placed in traditional cramps after final pressing in a hydraulic press (Linea H; Linea, Sao Paulo, SP, Brazil) under a load of 1,250 kgf for 5 minutes. In the RSFC groups (ND and RSD), trial packing was similar to the TFC method; however, during final pressing the flasks were positioned in the RSFC system [28]. During flask closure, the screws of the lower plate were fitted into the holes of the upper plate and, after applying flask pressure, the screw nuts were tightened to the screws. This procedure maintained constant flask closing pressure before release of the hydraulic press.

The flasks were immersed in water at room temperature, and the polymerizing unit (Termotron; Termotron Laboratory Products, Piracicaba, SP, Brazil) was programmed to raise the temperature to 74^0^C for 1 hour. This temperature was then maintained for 8 hours. Flask cooling was performed at room temperature before the acrylic resin bases were deflasked, and finished with abrasive stones (AcryPoint; Shofu Dental Corp, Menlo Park, Calf). Resin bases made by TFC-SD and RSFC-SD methods (n=5) were immersed individually in 150mL of distilled water in a glass container, and submitted to repeated simulated disinfections in a domestic microwave oven (Continental; Continental Domestic Lines, Manaus, AM, Brazil), calibrated to 650W for 3 minutes [23].Five simulated disinfections, one by day, were made in each denture base. During the interval between disinfection procedures, the denture base was stored in a stove (Orion 502; Fanem, Sao Paulo, SP, Brazil), immersed in distilled water at 37ºC. Control bases prepared by the TFC-ND and RSFC-ND methods (n=5) were not disinfected. All bases submitted to the ND and RSD treatments were attached to the corresponding stone casts with adhesive (Super Bonder; Loctite, Sao Paulo, SP, Brazil) placed on the ridge crest of the stone cast.

**Fig. (1):**
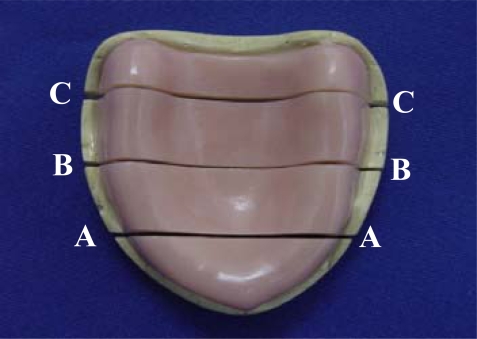
Transversal cuts in base-cast set: canine **(A)**, first molar **(B)**, and posterior palate **(C)**.

**Fig. (2):**
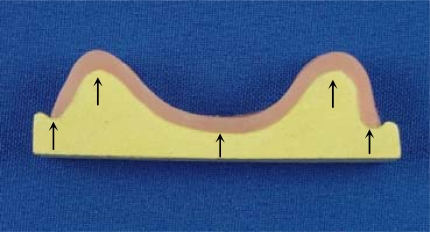
Measurement points corresponding to right and left residual ridge crests, midline, and right and left marginal limits of flanges (arrows).

The base-cast sets were transversally sectioned in a sawing device (Precat Manufacturing Co., Piracicaba, SP, Brazil) into 3 sections: canine, first molar, and posterior palate (Fig. **1**). The gap between the acrylic resin base and stone cast was measured in the 3 sections at 5 points, corresponding to the right and left residual ridge crests, the midline, and the right and left marginal limits of the flanges. Arithmetical mean of the reference points of each section was considered as the adaptation value for each section[27] (Fig. **2**). An optical micrometer (STM; Olympus Optical Co, Tokyo, Japan) with an accuracy of 0.0005mm was used for measurement purposes.

Data were submitted to 3-way analysis of variance (ANOVA), considering 3 factors (flask closure method, repeated simulated disinfection, and region) and their interactions. The split-plot design was used, supported by repeated measurements made from the same experimental group at different base-cast set cuts. Differences were submitted to multiple comparison testing (Tukey HSD test at α=.05).

## RESULTS

Three-way ANOVA (Table **1**) revealed significant difference in the denture adaptation for the variables flask closure (*P*<.001) and region (*P*<.001). The interactions between the factors were not significant.

Table **2** presents mean adaptation values for denture base obtained with TFC and RSFC methods. Denture base fit values obtained for the RSFC method (ND = 0.16 ± 0.05mm, and RSD = 0.17 ± 0.04mm) were significantly lower (*p*<.05) than those obtained for the TFC method (ND = 0.21 ± 0.05mm and RSD = 0.22 ± 0.05mm). RSD had no significant effect (*p*>.05) on the base adaptation when compared with the ND treatment.

Table **3** shows statistically significant difference (*p*<.05) in the adaptation among the regions for TFC method, when the denture bases were submitted to ND (canine: 0.15 ± 0.01mm, first molar: 0.20 ± 0.02mm, and posterior palate: 0.29 ± 0.01mm) or RSD (canine: 0.16 ± 0.01mm, first molar: 0.21 ± 0.01mm, and posterior palate: 0.30 ± 0.01mm) treatments. The best fit occurred in canine region, the worst was in posterior palate, and intermediary values were shown in first molar region. When the regions were compared individually, there was not statistically significant difference between ND and RSD treatments (*p*>.05).

Table **4** shows statistically significant difference (*p*<.05) in the adaptation among regions for RSD method, when the denture bases were submitted to ND (canine: 0.11 ± 0.01mm, first molar: 0.15 ± 0.03mm, and posterior palate: 0.22 ± 0.01mm) or RSD (canine: 0.12 ± 0.01mm, first molar: 0.18 ± 0.02mm, and posterior palate: 0.22 ± 0.01mm) treatments. The best fit occurred in canine region, the worst was in posterior palate, and intermediary values were shown in first molar region. When the regions were compared individually, there was not statistically significant difference between the ND and RSD treatments (*p*>.05). 

## DISCUSSION

The purpose of this investigation was to evaluate and compare the influence of repeated simulated disinfections by microwave energy on the adaptation of the maxillary denture base, using the TFC and RSFC methods. In the present *in vitro* study, the research hypothesis that the adaptation of the denture base could be adversely affected by RSD was not supported by the data. Three-way ANOVA revealed significant difference in denture adaptation for the variables closure and regions. The interactions between the factors were not significant (Table **1**).

Adaptation of the complete denture base may be affected by dimensional changes that may occur during or after base polymerization [2, 4, 6, 28].Linear shrinkage may also occur when the acrylic resin is polymerized by microwave irradiation, which is considered to be a dry heat polymerization method [1].

Improvement in the adaptation of the denture base showed in the TFC method using one only simulated disinfection by microwave was claimed to be related to the additional linear shrinkage that results from the residual polymerization of the acrylic resin [10].Conversely, in the present study, RSD by microwave energy had no influence on the adaptation of the denture base using either TFC or RSFC methods, when compared to those values obtained in the ND conditions (Table **2**).

Based on this study conditions, RSD by microwave irradiation did not cause any effect on the denture base adaptation in both TFC and RSFC methods. It is possible that the simulated microwave disinfection cycle used in this study had promoted different dimensional changes in the denture base, resulting in a stabilization of the base distortion. Probably, this base dimensional stability is due to balance of the shrinkage promoted by the microwave irradiation and stresses releasing during the water storage. In this case, the linear shrinkage due to residual polymerization was not sufficient to increase the adaptation level of the base in the ND conditions, as showed in earlier study [10].****However, the use of a microwave disinfection cycle with greater energy intensity and larger application time produced great discrepancy in the base adaptation to the stone cast [8]. Repeated microwave disinfections at 690W for 6 minutes promoted harmful to the adaptation of the denture bases [11].

When the flask closure methods were considered (Table **2**), the best base adaptation was observed with the RSFC method, with significantly different values when compared to the TFC method in both ND and RSD conditions. Studies reporting the influence of different post-pressing times (immediate, 6, 12 and 24 hours) used before acrylic resin polymerization on base accuracy [6], as well as different flask closure pressures [28] haveshown that adaptation improvement is also observed in denture bases prepared by the RSFC method when compared to those obtained by the TFC method.

RSD did not alter this finding, maintaining the denture base in better adaptation conditions, even when the flask closure was made with RSFC method. This result means that the denture base adaptation is not dependent of the flask closure method after RSD treatment, as well as it confirms that the dimensional change of the base probably occurs in the first simulated disinfection procedure [10]. In this present study, intermittent disinfections caused similar effects of shrinkage and expansion in the denture base, compensating the distortion effect occurred in the first SD procedure.

Despite the ability to produce a more accurate denture base in both ND and RSD procedures when compared to the TFC method, the RSFC method did not completely eliminate the dimensional changes that occurred during the denture base procedure.

When the region factor was analyzed in the TFC method (Table **3**), there was significant difference in the adaptation values among the canine, first molar, and posterior palate region. The tendency for differences in accuracy between regions has also been shown in earlier studies investigating flask closure methods [28], commercial brands of acrylic resins [4], and effect of delay prior to acrylic resin polymerization [6] as a result of the maxillary anatomy [10].

Better base adaptation in the canine region may be due to association between anatomic condition of the anterior region and acrylic resin polymerization shrinkage, where the stress released does not cause significant base distortion. In contrast, the anatomy of the posterior palate region allowed a large base distortion, causing greater base inaccuracy, whereas the first molar region shows intermediate dimensional change. Conversely, despite the linear shrinkage of the acrylic resin to cause significant effect on the base adaptation, and the internal stress release to produce dimensional changes in the acrylic resin [1], there was not statistically significant difference in each region, when the ND and RSD conditions were compared.

Similar results for TFC method were obtained in the RSFC method (Table **4**). These findings seem to confirm the different levels of the denture base adaptation in relation to different regions, independently of the studied variables. This fact shows the influence of the RSFC method in reducing the level of the base inaccuracy, which was not modified by the RSD procedure.

The present study did not show statistically significant difference on the base adaptation under RSD effect by microwave energy, when 5 repeated disinfections at 650W for 3 minutes were made with 24 hours interval and water storage at 37ºC during the intervals. Probably, the water immersion time was sufficient to compensate the dimensional change occurred during the first disinfection, showing conflicting results when compared to previous work using 690W for 6 minutes, and water storage for 7days between two disinfection procedures [9]. Probably, the conflicting findings are due to difference in the water storage period and/or potency and time of the microwave irradiancy used in the two studies.

The results of this study may be of clinical relevance when the denture bases were submitted to repeated microwave disinfection procedure as a clinic routine. On other hand, despite of this inaccuracy decrease between acrylic base and stone cast made by the RSFC method, the base adaptation remains still an inherent factor of the denture processing.

Although attempts were made to characterize the effect of RSD on denture base adaptation, this *in vitro* study is limited in predicting the effect of different microwave disinfection cycles. Further investigations are necessary to evaluate the effect of RSD on the denture adaptation in clinical use. Considering that the simulated microwave disinfection decreases the impact strength of the tooth/resin adhesion [29], and the effect on porosity appears only on the 2mm-thickness specimens [30], further studies are also necessary to verify the adhesion strength of the tooth-resin joint associate to mechanical retention of the tooth, commercial brands of acrylic resins, polymerization cycles, and denture base thickness.

## CONCLUSION

RSD did not significantly affect the base adaptation in the TFC and RSFC methods. In ND and RSD conditions, the base adaptation performed in the RSFC methods was significantly better when compared to the TFC.

1. The benefits of geriatric day hospital care have been controversial for many years.
2. In the TFC and RSFC methods, the base adaptation was significantly different among regions in either ND or RSD conditions. In each region, the base adaptation performed in the TFC and RSFC methods was statistically similar when the ND and RSD conditions were compared

## Figures and Tables

**Table 1.** Results of 3-Way ANOVA

Variation Cause	*df*	Sum of Squares	Mean Square	F	*P*
Closure	1	0.039	0.039	83.857	.001
Disinfection	1	0.001	0.001	3.756	.005
Closure x Disinfection	1	0.000	0.000	0.001	.973
Error A	16	0.013	0.000		
Repetition	19	0.043			
Region	2	0.148	0.074	156.113	.001
Closure x Region	2	0.002	0.001	2.561	.085
Disinfection x Region	2	0.000	0.000	0.508	.609
Clos. x Disin. x Reg.	2	0.000	0.000	0.327	.727
Error (B)	32	0.005	0.000		
Total	59	0.171			

General mean = 0.19; variation coefficient (A) = 9.374%; variation coefficient (B) = 7.049%

**Table 2.** Means Values (mm) for Denture Base Adaptation Concerning to Flask Closure Method and Microwave Disinfection (Standard Deviations are Given in Parenthesis)

Flask Closure	Microwave Disinfection	
	Non-Disinfected	Simulated
TFC	0.21 (0.05) a A	0.22 (0.05) a A
RSFC	0.16 (0.05) b A	0.17 (0.04) b A

Means values followed by different lower case letters in each column and upper case letters in each row differ significantly at 5%

**Table 3.** Means Values (mm) for Denture Base Adaptation Concerning to Cut Region and Microwave Disinfec-tion for TFC Method (Standard Deviations are Given in Parenthesis)

Region	Microwave Disinfection	
	Non-Disinfected	Simulated
Canine	0.15 (0.01) a A	0.16 (0.01) a A
First molar	0.20 (0.02) b A	0.21 (0.01) b A
Posterior palate	0.29 (0.01) c A	0.30 (0.01) c A

Means values followed by different lower case letters in each column and upper case letters in each row differ significantly at 5%

**Table 4.** Means Values (mm) for Denture Base Adaptation Concerning to Cut Region and Microwave Disinfection for RSFC Method (Standard Deviations are Given in Parenthesis)

Region	Microwave Disinfection	
	Non-Disinfected	Simulated
Canine	0.11 (0.01) a A	0.12 (0.01) a A
First molar	0.15 (0.03) b A	0.18 (0.02) b A
Posterior palate	0.22 (0.01) c A	0.22 (0.01) c A

Means values followed by different lower case letters in each column and upper case letters in each row differ significantly at 5%
